# Chromobox homolog 8 (CBX8) Interacts with Y-Box binding protein 1 (YBX1) to promote cellular proliferation in hepatocellular carcinoma cells

**DOI:** 10.18632/aging.102241

**Published:** 2019-09-08

**Authors:** Lushan Xiao, Zixiao Zhou, Wenwen Li, Jie Peng, Qingcan Sun, Hongbo Zhu, Yang Song, Jin-Lin Hou, Jingyuan Sun, Hui-Chuan Cao, Dong Zhongyi, Dehua Wu, Li Liu

**Affiliations:** 1 State Key Laboratory of Organ Failure Research, Nan Fang Hospital, Southern Medical University, Guangzhou 510515, China; 2 Guangdong Provincial Key Laboratory of Viral Hepatitis Research, Nan Fang Hospital, Southern Medical University, Guangzhou 510515, China; 3 Department of Infectious Diseases, Nan fang Hospital, Southern Medical University, Guangzhou 510515, China; 4 Department of Radiation Oncology, Nan fang Hospital, Southern Medical University, Guangzhou 510515, China; 5 Department of Oncology, The Second Affiliated Hospital, Guizhou Medical University, Kaili, P.R. China; 6 Department of Medical Oncology, The First Affiliated Hospital of University of South China, Hengyang, Hunan 421001, P.R. China

**Keywords:** HCC, CBX8, YBX1, cyclin D1, HCC proliferation

## Abstract

Polycomb group (PcG) proteins have recently been identified as critical regulators in tumor initiation and development. However, the function of CBX8 in human hepatocellular carcinoma (HCC) remains largely unknown. Our study was designed to explore the biological function and clinical implication of CBX8 in HCC. We investigated the interplay between CBX8 and cell cycle through Gene Set Enrichment Analysis and western blotting. Bioinformatics tools and co-immunoprecipitation were used to explore cell cycle regulation. Finally, we studied the expression and clinical significance of CBX8 in HCC through 3 independent datasets. CBX8 was upregulated in HCC and its expression correlated with cell cycle progression. CyclinD1 was downregulated by CBX8 knockdown but upregulated by CBX8 overexpression. YBX1 interacted with CBX8 and regulated the cell cycle. Moreover, targeting YBX1 with specific siRNA impaired CBX8-mediated regulation of CyclinD1. CBX8 overexpression boosted HCC cell growth, while CBX8 knockdown suppressed cell proliferation. Further, YBX1 interacted with CBX8. YBX1 knockdown compromised the proliferation of CBX8 overexpressing cells. CBX8 promotes HCC cell proliferation through YBX1 mediated cell cycle progression and is related to poor HCC prognoses. Therefore, CBX8 may serve as a potential target for the diagnosis and treatment of HCC.

## INTRODUCTION

Liver cancer was predicted to be the sixth most commonly diagnosed cancer and the fourth leading cause of cancer-related mortality worldwide in 2018, with about 841,000 new cases and 782,000 deaths annually [1]. Hepatocellular carcinoma (HCC), accounting for most (75%–85%) of the primary liver cancers occurring worldwide, remains one of the most prevalent and deadliest human cancers [1]. Despite improvements in surgical resection, liver transplantation, molecular-targeted therapy and immunotherapy, the prognosis of HCC has still remained poor over the last decades [2–4]. HCC is typically diagnosed at an advanced stage and has shown a high recurrence rate, resulting in adverse outcomes in patients [5]. Therefore, it is critical to clarify the molecular mechanisms underlying HCC progression and identify novel therapeutic targets for HCC treatment.

Among pathways and factors involved in the formation and maintenance, the PcG proteins have garnered the attention of researchers [6, 7]. Deregulation and dysfunction of PcG proteins often lead to blockade or inappropriate activation of developmental pathways, enhancement of cellular proliferation, inhibition of apoptosis, and restoration of the cancer stem cell population [7–10]. PcG proteins function principally as two large multisubunit complexes, Polycomb repressive complex 1 (PRC1) and Polycomb repressive complex 2 (PRC2) [7]. Under normal circumstances, PRC1 maintains the histone methylation induced by PRC2 to pass on the inactivation signals where CBX8, also known as human Polycomb 3 (HPC3), functions as a transcriptional repressor as part of PRC1 [7]. CBX8 was shown to inhibit the expression of INK4a/ARF to bypass cell senescence in fibroblasts [11]. PcG proteins have been also shown to exhibit non-polycomb functions, contributing to the regulation of diverse cellular functions [7, 12–14]. Recently, CBX8 has been demonstrated to exhibit oncogenic functions in a non-canonical manner in human malignancies. For instance, Cbx8 acts non-canonically with Wdr5 to promote mammary tumorigenesis [15]. Recently, abnormal expression of CBX8 in multiple tumors was reported. CBX8 play different roles in different tumors [6, 15–21]. However, the mechanism by which CBX8 regulates the malignant growth of HCC remains unclear.

In our study, we aim to explore the expression of CBX8 in HCC and its clinical significance by gene microarrays and bioinformatics tools. To further explore the mechanism by which CBX8 plays a role in HCC, we examined the direct coupling of proteins through bioinformatics tools and co-immunoprecipitation (Co-IP) experiments. Finally, we investigated the biological function of CBX8 in HCC cells through CCK-8, and EdU experiments. We set out to identify the mechanism by which CBX8 regulates the malignant progression of HCC. An additional goal is to understand whether CBX8 can be used as a target to block the malignant progression of HCC.

## RESULTS

### CBX8 is positively correlated with cell cycle signaling pathway

**Figure 1:**
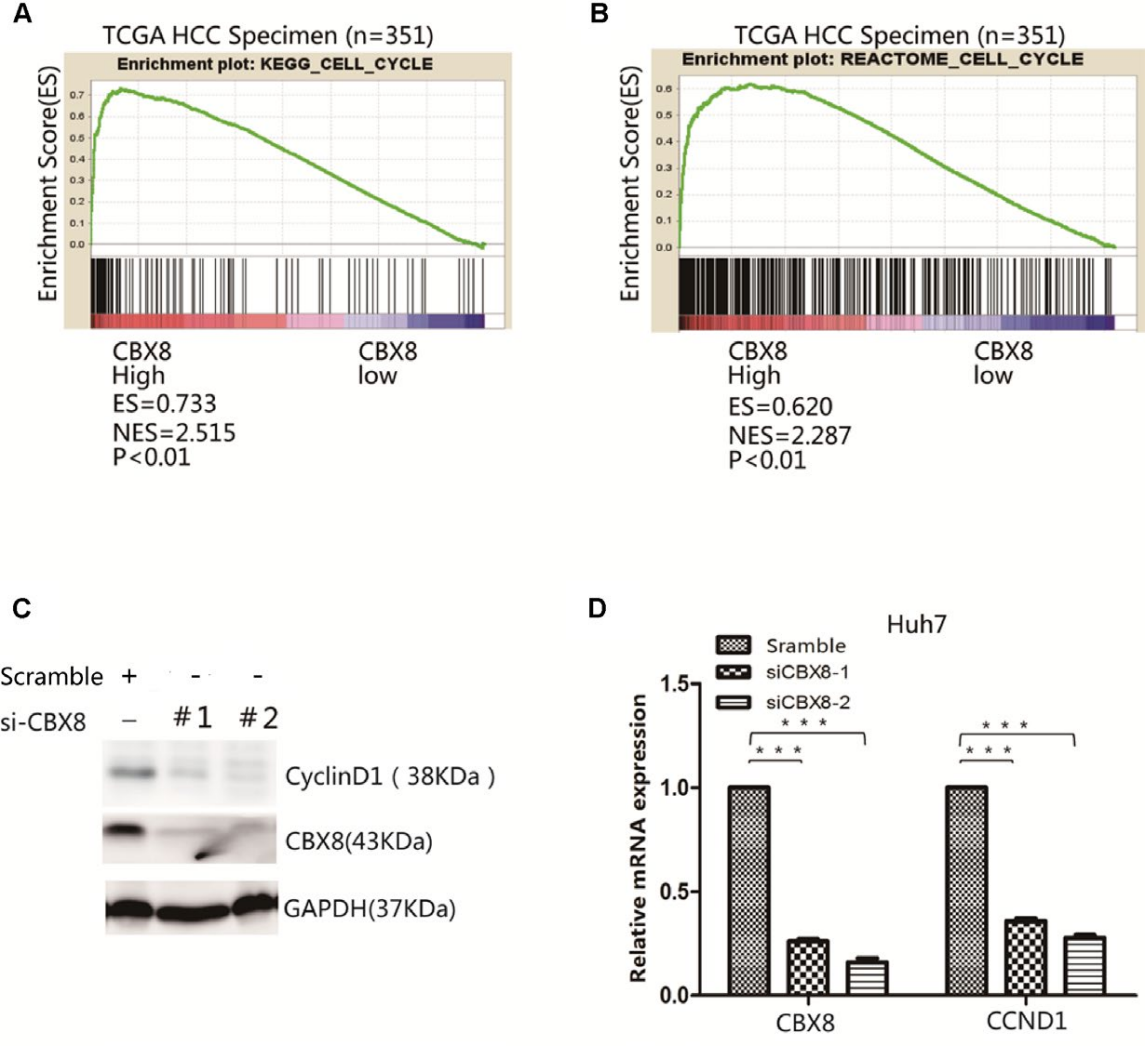
**CBX8 is positively correlated with cell cycle signaling pathway.** (**A**–**B**) Results of Gene Set Enrichment Analysis (GSEA) were plotted to visualize the correlations between CBX8 cell cycle gene signatures in the TCGA cohort (P < 0.01). (**C**–**D**) Both protein and mRNA levels of CyclinD1 in CBX8 Knockdown Huh7 cells, as detected by western blotting and q-RT-PCR.

To dissect the molecular mechanism of CBX8, we carried out Gene Set Enrichment Analysis (GSEA) of The Cancer Genome Atlas (TCGA) cohort and found that CBX8 high expressing groups were enriched for cell cycle-related gene sets (Figure 1A and 1B). To confirm its function in regulating the cell cycle, we detected a series of cell cycle-related genes after interfering with CBX8 and found that CCND1 was significantly down-regulated (Figure 1D, Supplementary Figure 1A), and that CyclinD1 was down-regulated by CBX8-knockdown at the protein level (Figure 1C).

### CBX8 interact with YBX1

**Figure 2:**
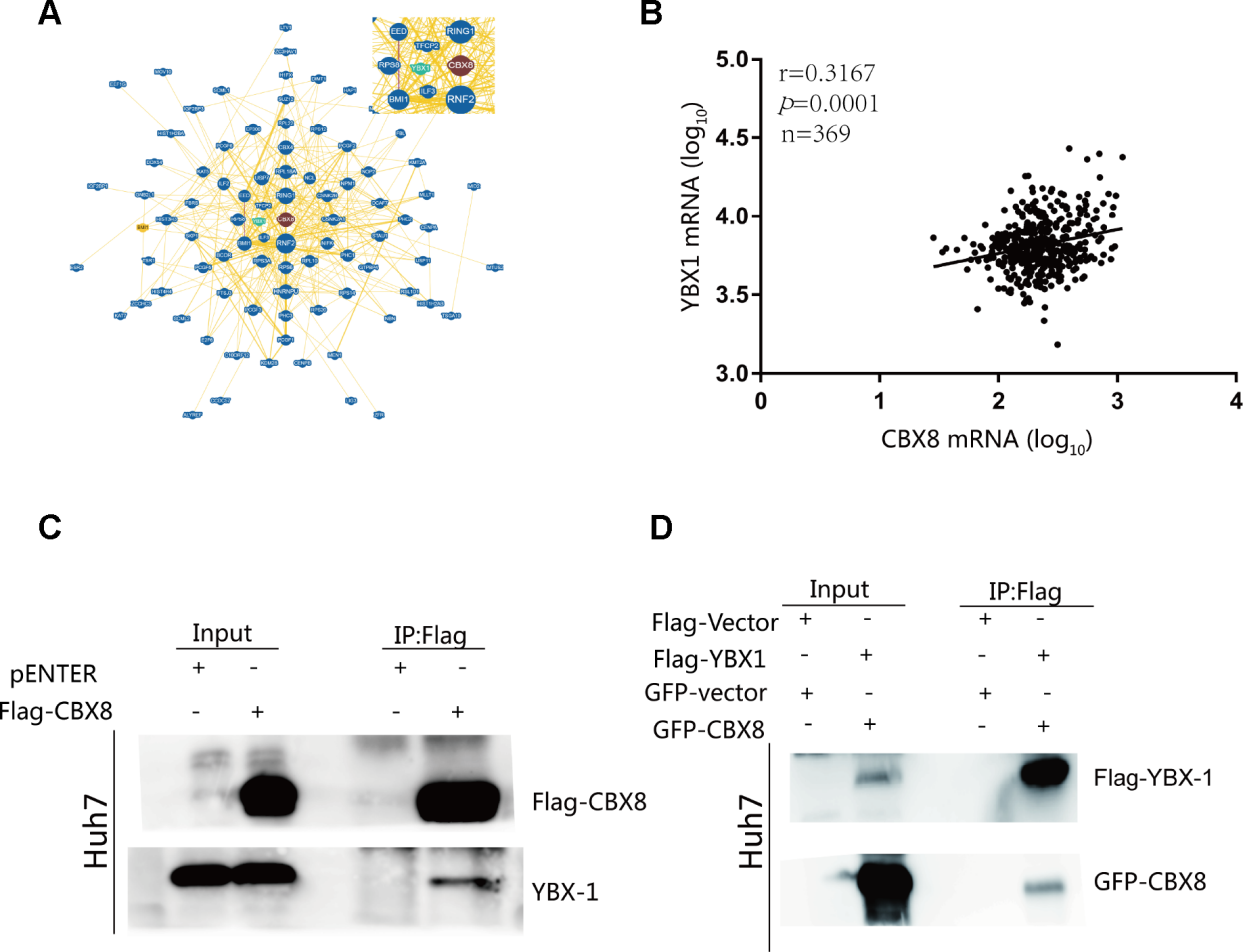
CBX8 interacts with YBX1 (**A**) CBX8 may interact with YBX1, as visualized by BioGRID. (**B**) Correlation between CBX8 mRNA and YBX1 mRNA in TCGA cohort (P = 0.0001). (**C**) Huh7 cells were transfected with Flag-CBX8 overexpression vector for 48 h. An immunoprecipitation (IP) assay, using an anti-Flag antibody, was used to detect the binding of CBX8 and YBX1. (**D**) Huh7 cells were transfected with GFP-CBX8 and Flag-YBX1 overexpression vector for 48 h. An IP assay, using an anti-Flag antibody, was used to detect the binding of CBX8 and YBX1.

In order to unveil the mechanism underlying the oncogenic function of CBX8 in modulating the cell cycle, we searched for proteins predicted to interact with CBX8 through BioGRID (https://thebiogrid.org/) as well as in the literature, and found that CBX8 may bind to YBX1, ILF3(Interleukin Enhancer Binding Factor 3), TFCP2 (Transcription Factor CP2) and UBE2S (Ubiquitin Conjugating Enzyme E2S) (Figure 2A) [23]. To confirm their interaction, we first tested the correlation between their mRNA expression levels in HCC. As detected in TCGA cohort, CBX8 expression was positively correlated with YBX1 and UBE2S mRNA levels (Figure 2B and Supplementary Figure 2A–2C) (r = 0.3167; P< 0.001 and r=0.3190; P=0.0001). More importantly, it was demonstrated via co-immunoprecipitation (Co-IP) experiments that CBX8 interacted with YBX1 (Figure 2C–2D), rather than with ILF3, TFCP, UBE2S (Supplementary Figure 2D).

### CBX8 increase levels of CyclinD1 through YBX1

**Figure 3:**
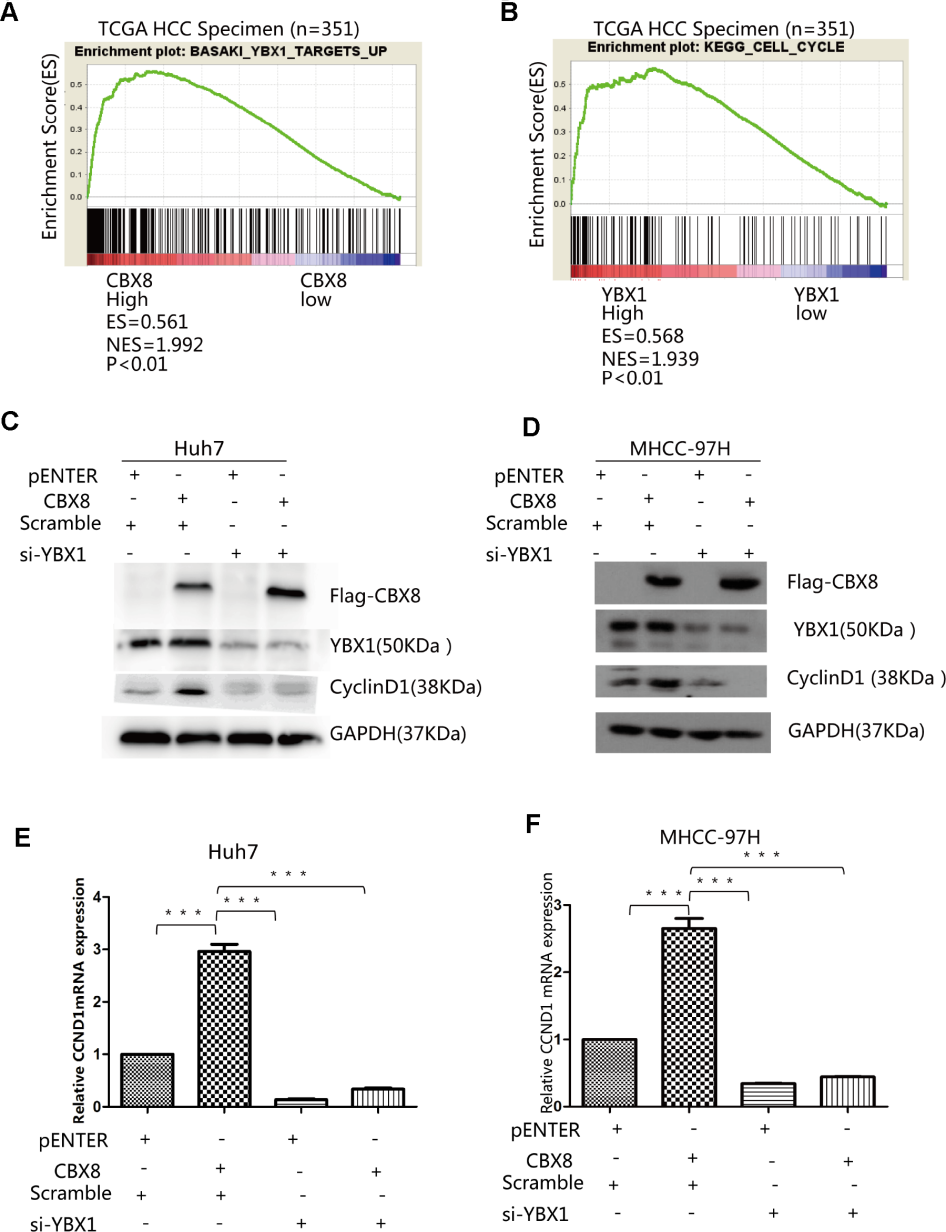
**CBX8 increase levels of CyclinD1 through YBX1.** Results of GSEA were plotted to visualize the correlation between the expression of CBX8 (**A**) or YBX1 (**B**) and gene signatures of YBX1 target up or KEGG cell cycle in the TCGA cohort (P < 0.01). (**C**–**F**) Both protein and mRNA levels of CyclinD1 in Huh7 and MHCC-97H cells-overexpressing CBX8 or YBX1 knock-down as detected by western blotting and q-RT-PCR.

We explored whether CBX8 can play a role in promoting the cell cycle through YBX1 in HCC cells. Firstly, GSEA results showed that YBX1 targets were enriched in high CBX8 expressing groups (Figure 3A) and YBX1 expression was positively correlated with the cell cycle related genes (Figure 3B). To confirm the association of CBX8 with YBX1 and the cell cycle, we transfected a plasmid containing the CBX8 sequence in Huh7, MHCC-97H and SK-Hep-1 cells, and found that CBX8 overexpression markedly increased the level of CyclinD1, while knockdown of YBX1 with a specific siRNA abrogated its effect (Figure 3C–3D, Supplementary Figure 3A). Moreover, CCND1, the mRNA encoding for CyclinD1, was also regulated by CBX8 and YBX1 in the same manner (Figure 3E–3F, Supplementary Figure 3B). Moreover, when we interfered with CBX8 and simultaneously overexpressed YBX1 in Huh7 cells, YBX1 rescued the down-regulation of CBX8-induced CCND1 down-regulation (Supplementary Figure 3C). Therefore, CBX8 promotes the expression of CyclinD1 in a YBX1-dependent manner.

### CBX8 promotes HCC cell proliferation in vitro and in vivo

**Figure 4:**
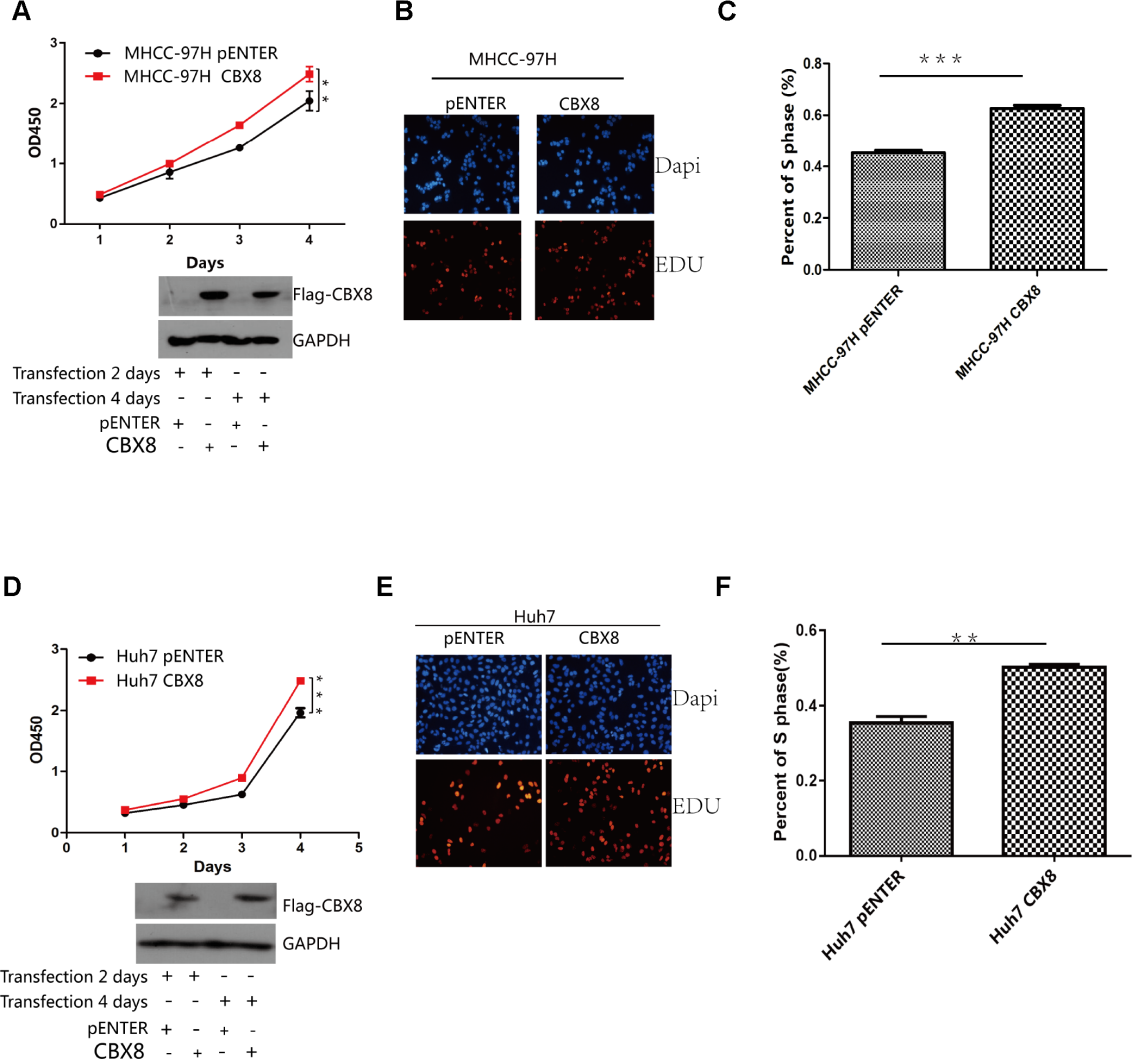
**CBX8 overexpression promotes cell proliferation.** (**A** and **D**) Cell proliferation was evaluated by the CCK-8 assay. (**B**–**F**) Effect of CBX8 on cell proliferation as measured by EdU assays *P < 0.05; **P < 0.01;***P < 0.001.

Next, whether CBX8 plays a role in the cell proliferation was evaluated. As depicted by CCK-8 and EdU assay, MHCC-97H cells overexpressing CBX8 showed enhanced proliferation compared with the control group (Figure 4A–4C). Another two HCC cell lines, Huh7 and SK-Hep-1 also showed increased cell growth after overexpressing CBX8 (Figure 4D–4F, Supplementary Figure 4A–4C).

**Figure 5:**
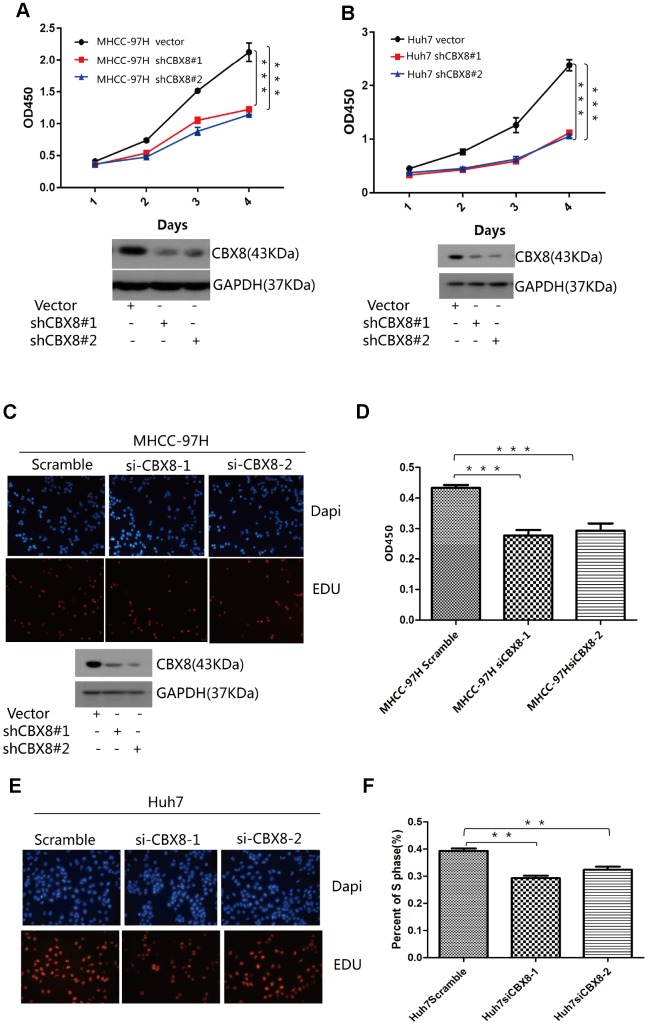
**Knockdown of CBX8 dramatically decreases the cell proliferation in vitro.** (**A** and **B**) Cell proliferation was evaluated by the CCK-8 assay. (**C**–**F**) Effect of CBX8 on cell proliferation as measured by EdU assays *P < 0.05; **P < 0.01; ***P < 0.001.

**Figure 6:**
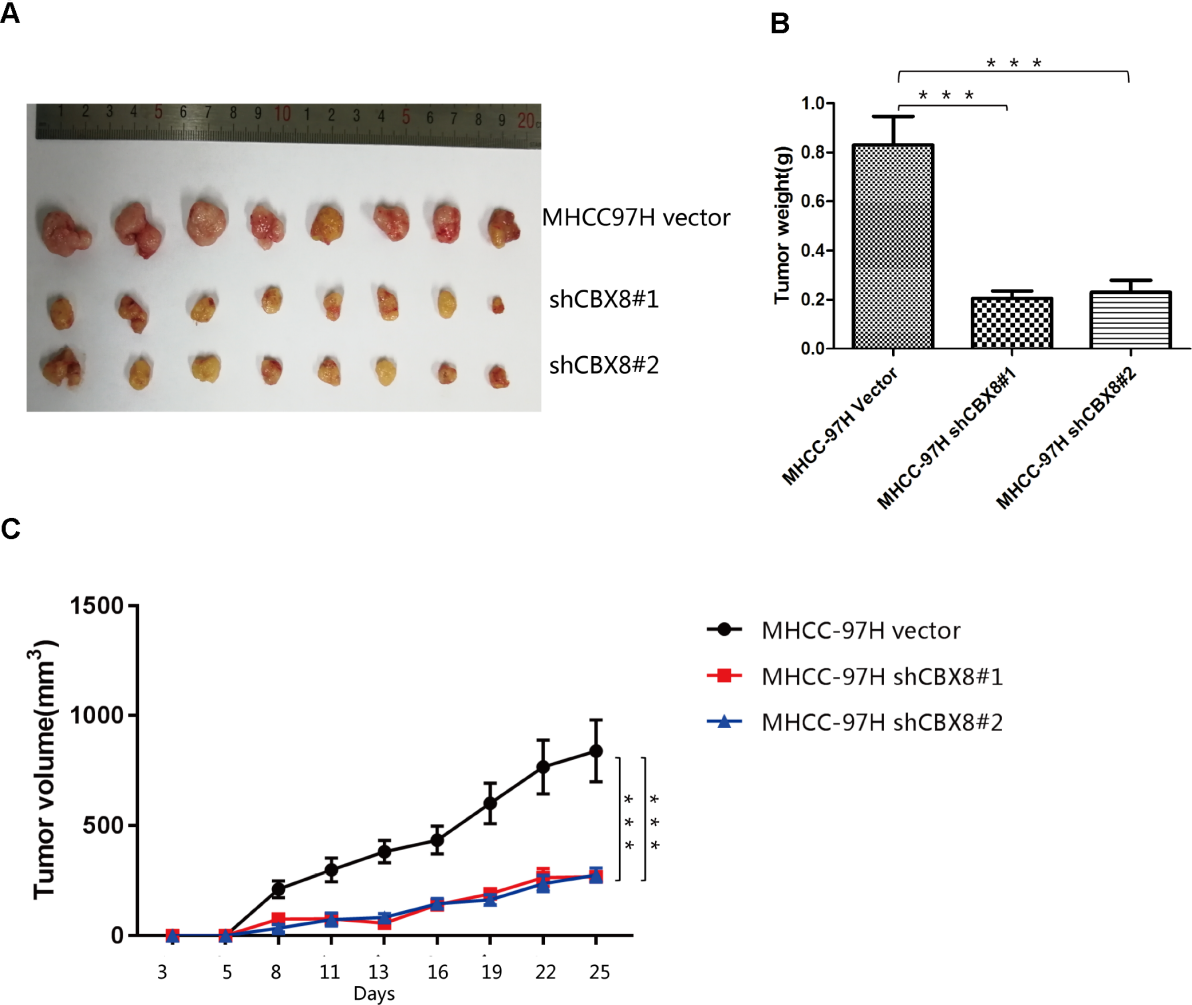
Knockdown of CBX8 dramatically decreases cell proliferation in vivo (**A**) Representative images of tumors formed in nude mice subcutaneously injected with CBX8–silenced MHCC-97H cells. (**B**) Tumor weights (***P <0.001). (**C**) Tumors induced by CBX8 silencing in MHCC-97H cells (***P <0.001) showed markedly lower growth rates than the control cells.

To further confirm the regulatory function of CBX8 in the cell proliferation, we knocked down CBX8 and detected the changes of proliferation in MHCC-97H, Huh7 and SK-Hep-1 cells. In three cell lines, knockdown of CBX8 reduced the proliferation rate as compared with the control groups (Figure 5A–5B, Supplementary Figure 5A–5C). Results of EdU assay revealed that cells with CBX8 knockdown were stagnated before S phase of the cell cycle (Figure 5C–5F, Supplementary Figure 5D–5E). Furthermore, the effect of CBX8 knockdown on tumor growth in vivo was examined using mouse subcutaneous xenograft models. Tumor xenografts derived from CBX8-knockdown MHCC-97H and SK-Hep-1 cells exhibited smaller volumes, lower weights, and formed more slowly than tumors obtained from the control cells (Figure 6A–6C and Supplementary Figure 6A–6D). In summary, CBX8 promotes HCC cell proliferation in vitro and in vivo.

### CBX8 promotes HCC cell proliferation through YBX1

**Figure 7:**
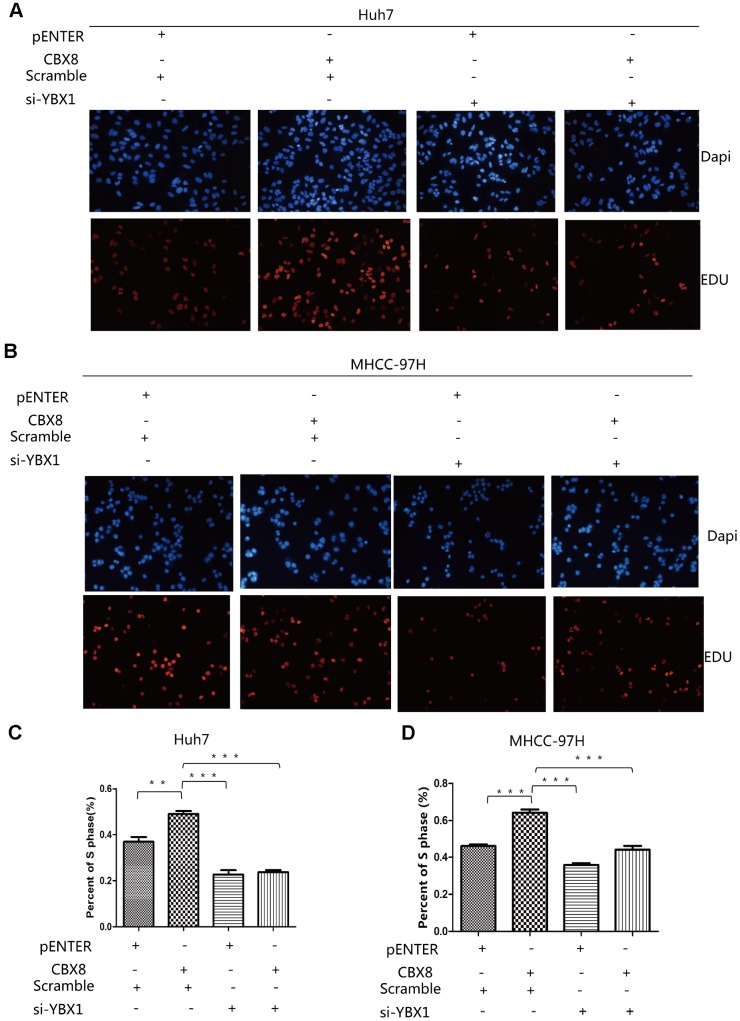
**CBX8 promotes HCC cell proliferation through YBX1.** (**A**–**D**) Cell proliferation, as detected by EdU assays **P< 0.01; ***P < 0.001.

Given that CBX8 regulated CyclinD1 expression through YBX1, we hypothesize that its regulatory function in cell proliferation is YBX1-dependent. We repeated the EdU assay in Huh7, MHCC-97H and SK-Hep-1 cells overexpressing CBX8 with or without simultaneous knockdown of YBX1 with targeted siRNA. Results showed that the increased proliferation of Huh7, MHCC-97H and SK-Hep-1 cells induced by CBX8 overexpression was attenuated by interfering YBX1 (Figure 7A–7D, Supplementary Figure 7A, 7C). Furthermore, when we interfered with CBX8 and simultaneously overexpressed YBX1 in Huh7 cells, YBX1 rescued the effect of CBX8 down-regulation on the inhibition of cell proliferation (Supplementary Figure 7B, 7D). These results confirm that CBX8 promoted cell proliferation in a YBX1-dependent manner.

### CBX8 expression is up-regulated in HCC and is correlated with prognosis

**Figure 8:**
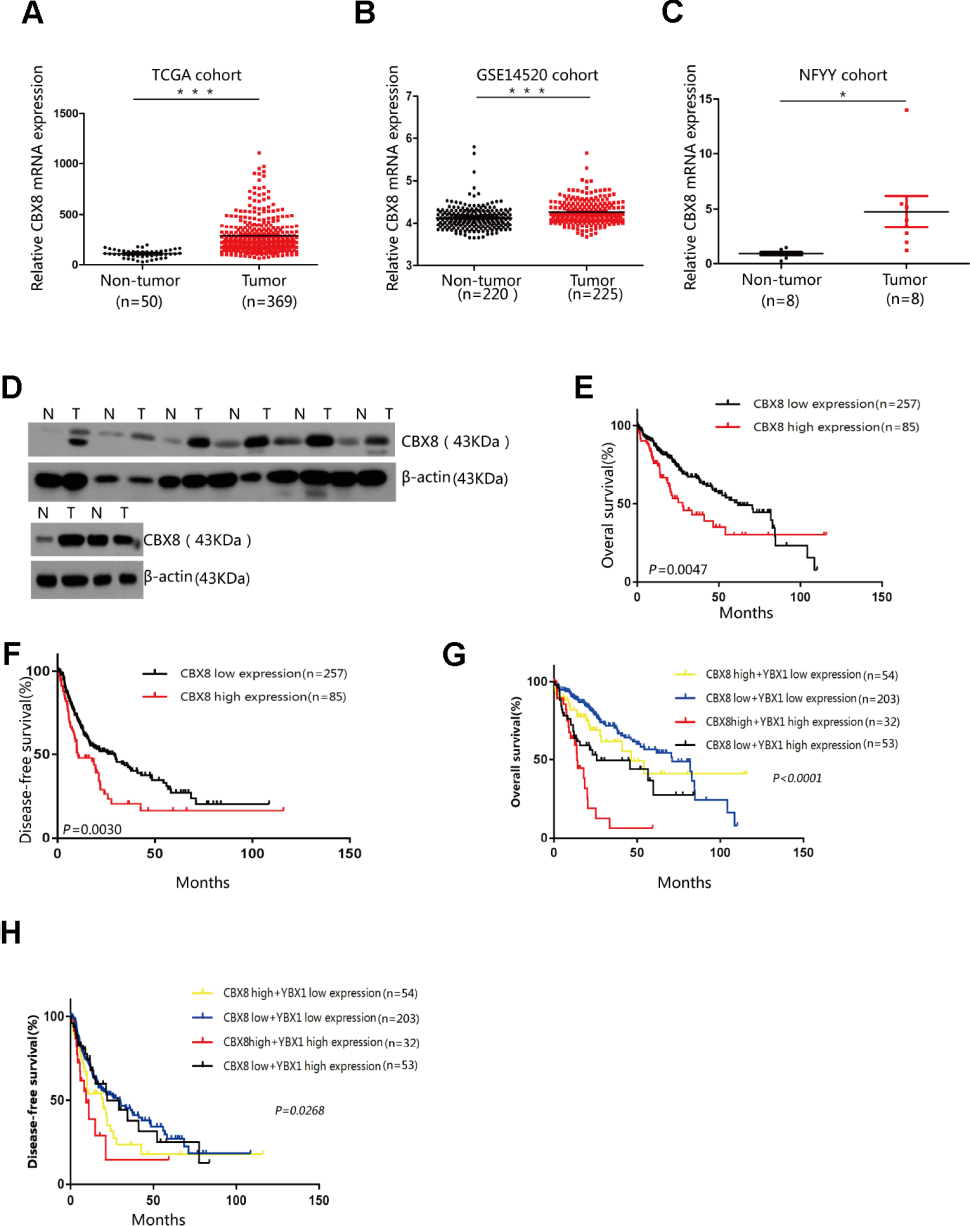
**CBX8 expression is up-regulated in HCC and is correlated with poor prognosis.** (**A**–**B**) Expression of CBX8 in the TCGA and GSE14520 cohorts (P < 0.001) (**C**) Expression levels of CBX8 in HCC tissues and adjacent non-tumor tissues (NFYY cohort) as measured by qRT-PCR analysis (P < 0.05); β-actin was used as an internal control. Data are shown as median with interquartile range. (**D**) The protein expression of CBX8 in 8 HCC samples was determined by western blotting. (**E**, **F**) Kaplan-Meier analysis of the overall and disease-free survival in the TCGA cohort based on CBX8 expression (**G**, **H**) Kaplan-Meier analysis of the overall and disease-free survival in the TCGA cohort based on CBX8 and YBX1 expression.

As CBX8 plays a significant role in cell proliferation, we next explored its clinical importance. Clinical data of a cohort containing 369 HCC cases and 50 non-tumor cases were obtained from TCGA website (https://www.cancer.gov), and analysis revealed that CBX8 was upregulated in HCC lesions when compared with normal tissues (Figure 8A). We also analyzed another cohort (GSE14520) from GEO database and discovered that in comparison with non-tumor tissues, HCC lesions exhibited higher levels of CBX8 mRNA (Figure 8B). To confirm these results, we compared its expression in 8 pairs of HCC tissues and adjacent normal liver tissues. Accordingly, CBX8 was overexpressed at both mRNA and protein levels in the tumor (Figure 8C–8D). Not only in HCC, but CBX8 expression was increased in pan-cancer as compared with the corresponding non-tumor tissues (Supplementary Figure 8A).

Then, we sought to determine the clinical significance of CBX8 and performed a series of Kaplan-Meier survival analyses. In the TCGA cohort, patients with high CBX8 expression suffered shortened overall survival and disease-free survival (Figure 8E–8F). We further grouped these patients with different pathological features followed by survival analyses. Results showed that CBX8 was of significant prognostic value in HCC patients at the early and especially the advanced stages (Supplementary Figure 9A–9B). While HCC is often developed from an infection of hepatitis virus or alcohol abuse, CBX8 expression was also potent in clarifying prognosis in patients with viral or toxic pathogeny (Supplementary Figure 9C–9F). Nonetheless, high CBX8 expression indicated poor prognosis in patients with vascular invasion rather than in those without (Supplementary Figure 9G–9H). Furthermore, in patients undergoing sorafenib treatment, CBX8 expression was also a factor markedly effecting their survival (Supplementary Figure 9I). Our current data indicated that CBX8 regulates cell proliferation through YBX1, and patients with simultaneous high CBX8 and YBX1 expression also had shorter survival and poorer prognosis (Figure 8G–8H). YBX1 was also upregulated in HCC tissues (Supplementary Figure 10A–10B), YBX1 was only capable of predicting overall survival but incapable of disease-free survival (Supplementary Figure 10C–10D). However, YBX1 expression could distinguish between the overall survival curves of patients with various pathological features (Supplementary Figure 11A–11I).

## DISCUSSION

The key finding of the current study was that CBX8 promotes cell proliferation and the cell-cycle progression by interacting with YBX1. Our results showed that CBX8 is overexpressed in HCC tissues as compared to corresponding adjacent non-tumor tissues from 3 independent cohorts. Moreover, patients with simultaneous overexpression of CBX8 and YBX1 featured shorter survival and poorer prognosis. Thus, our results indicated that CBX8 may serve as a potential target for the diagnosis and treatment of patients with HCC.

Dysregulation of CBX8 has been reported in various human cancers [15, 16, 20–22, 24]. Growing research has shown the cell proliferation promoting effects of CBX8 in different types of cancers [15, 18, 20]. Unlashed cell proliferation is the hallmark of cancer, and tumor cells have typically acquired damage of genes that directly regulate their cell cycles [25, 26]. Genetic alterations affecting Cyclin D1, and control exit from the G1 phase of the cell cycle, are frequently documented in human cancers and inactivation of this pathway may as well be necessary for tumor development [25, 27]. Interestingly, we found that CBX8 is involved in the cell cycle signaling pathway and regulates the expression of CyclinD1 in HCC cells.

Also, our research found that CBX8 regulated the cell cycle through YBX1. The Y-box binding protein (YBX1) is known to be a multifunctional transcription and translation factor regulating protein expression; its aberrant activation thus influences various malignant phenotypes of cancer cells [28–32]. For example, it is involved in promoting cancer progression, cell proliferation and multidrug resistance [29, 32–34]. Our study uncovered its interaction with CBX8. Their interaction was predicted by bioinformatics tools and further confirmed with Co-IP and other experiments. Moreover, the function of CBX8 in driving the cell cycle progression and cell proliferation was impaired upon YBX1 knockdown. Therefore, we summarized that CBX8 regulates the cell cycle progression through YBX1 in HCC.

We also found distinctive upregulation of CBX8 in fresh tissues, identified by qRT-PCR and western blot analyses. In TCGA cohorts, patients with high CBX8 expression experienced significantly shorter overall and disease-free survival, as compared with the low CBX8 expression group. Taken together, these findings suggested the misregulation of CBX8 is a potential biomarker for clinical surveillance of tumor progression. Zhang’s and Bo Tang’s reports are consistent with our results and have further confirmed the reliability of our results. However, the mechanisms of malignant HCC progression are different [18, 22]. We first found a new important binding partner for CBX8, and found that CBX8 regulates the proliferation of HCC cells through the regulation of cell cycle pathways. Moreover, we discovered that CBX8 represent a better predictor for poor prognosis of tumors, especially in case of staging, co-occurrence with hepatitis, and when the tumor is treated with sorafenib. More importantly, we found that the simultaneous overexpression of CBX8 and YBX1 is an excellent predictor of poor prognosis in HCC cases. This novel marker may serve as a potential target for the diagnosis and treatment of patients with HCC in the future.

In summary, we identified CBX8 as an oncogene with prognostic significance in HCC. CBX8 promotes HCC progression through activating the expression of CyclinD1 by binding to YBX1. Our study provides, for further evaluation, a novel therapeutic target for hepatocellular carcinoma treatment. However, our research also has some limitations, for example, the lack of multi-center and large sample sets for verification, limits the validity of our findings. In the future, more clinical samples will be used to verify the results. Moreover, the mechanisms surrounding CBX8 need further exploration, such as the mechanisms by which CBX8 affects CyclinD1 through YBX1. Of note, synthetic inhibitors are also essential tools to explore the role of CBX8 in HCC. For instance, inhibitors of EZH2, a vital member of the PRC2 complex, play a role in multiple tumors, and specific inhibitors are currently under clinical trial [35–40].

## MATERIALS AND METHODS

### Human HCC samples

“NFYY cohort”: HCC samples and paired noncancerous specimens from 8 HCC patients who underwent hepatectomies between January 2012 and January 2013 at Nan fang Hospital, Southern Medical University (Guangzhou, China) were included in this study. Tissues were frozen with liquid nitrogen instantly after hepatectomies and stored in a refrigerator at −80°C. The study was reviewed and approved by the Nan fang Hospital Institutional Review Board.

### Cell lines

SK-Hep-1, Huh7 and MHCC-97H cells were obtained from the Cell Bank of Type Culture Collection (Chinese Academy of Sciences, Shanghai, China). Cells were cultured under the conditions advised by the supplier.

### Animal studies

A subcutaneous xenograft model was established to determine the effect of CBX8 on tumor growth in vivo. BALB/c nude mice (males, 4–5-weeks-old) were purchased from the Central Laboratory of Animal Science, Southern Medical University (Guangzhou, China). Experimental procedures in this study were performed according to our institutional guidelines for using laboratory animals and were approved by the Institutional Animal Care and Use Committee of Nanfang Hospital. In total, 1 × 107 MHCC-97H, SK-Hep-1 stably expressing sh-CBX8, and vector control were subcutaneously injected into the flank of the mice (Genechem Company Ltd., Shanghai, China). After 25 days, the mice were sacrificed. Tumor growth was examined every 2-3 days. Tumors were weighed after removal.

### Bioinformatics analysis

“TCGA cohort”: A cohort from the TCGA (https://tcga-data.nci.nih.gov/tcga/) database including 369 HCC patients with CBX8 and YBX1 as well as follow-up information was included in our study to explore the expression levels of CBX8 and YBX1 in HCC. (Overall survival) OS and (Disease-free survival) DFS were also assessed according to CBX8 or YBX1 expression levels as follows: patients were split into two groups on the basis of CBX8- expression status in the primary tumor. Those with a CBX8-expression level ranked in the top quartile were classified into the high expression group and the rest into the low expression group [41–43].

“GSE14520 cohorts”: microarray data (GEO accession numbers GSE14520 were down loaded from the GEO (http://www.ncbi.nlm.nih.gov/geo/) to validate CBX8 or YBX1 expression level in HCC. [24] GCBI (Gene-Cloud of Biotechnology Information) (Shanghai, China, https://www.gcbi.com.cn) is a data analysis website. In this study, we used GCBI to identify relative CBX8 mRNA expression in human cancers. Stratified analysis showed the correlation of CBX8 or YBX1 and survival in liver cancers was analyzed in Kaplan–Meier Plotter (http://kmplot.com/analysis/) [44].

CCK-8 and EdU Assays, qRT-PCR, Plasmid Construction and Transient Transfection, Western Blot, RNA interference, Co-Immunoprecipitation (Co-IP), Lentiviral Construction and Cell Transfection; these were performed as described previously and are detailed in the Supplementary Methods.

### Statistical analysis

Data are presented as the mean ± standard error of the mean (SEM) from three independent experiments, except when indicated otherwise. The t-test or one-way analysis of variance (ANOVA) for comparison of two groups, multi-way classification ANOVA for cell proliferation, Pearson’s correlation for analyzing the correlation between CBX8 and YBX1, and Kaplan-Meier for the survival analysis were used in this study. Statistical analyses were performed using SPSS 21.0 software (Abbott Laboratories, North Chicago, IL, USA). A P < 0.05 (two-tailed) was considered statistically significant.

## Supplementary Material

Supplementary Methods

Supplementary Figures

Supplementary Tables
