# Supplementary material for: Chromobox homolog 8 (CBX8) Interacts with Y-Box binding protein 1 (YBX1) to promote cellular proliferation in hepatocellular carcinoma cells
Source: Aging (Albany NY). 2019 Sep 8;11(17):7123–49. doi: 10.18632/aging.102241 (PMC6756871; doi:10.18632/aging.102241)
Supplement: Supplementary Methods [file aging-11-102241-s001.pdf]

SUPPLEMENTARY METHODS

Plasmid construction and transient transfection

Flag-CBX8 plasmid (Catalog Number: CH853665) was purchased from Shandong Wei Zhen Biotechnology Co. Ltd (China). Flag-YBX1 and GFP-CBX8 were purchased from Shanghai Ji Kai Biotechnology Co., Ltd (China). Cells seeded overnight were transiently transfected with plasmids or control vectors (empty vectors) at 90% cell confluency using Lipofectamine 3000 (Invitrogen, Carlsbad, CA). After 48 h, cells were harvested for the following assays.

Cell counting kit-8 (CCK-8) assays

Cellular proliferation capacity was tested with Cell Counting Kit 8 (CCK-8) assay (Dojindo, Kumamoto, Japan) following the manufacturer's protocol. 1×103 cells per well were seeded in 96-well plate and grown for the given time points. 24 hours later, 10μl CCK-8 reagent was added to each plate, and cultured for another 2 hours at 37 °C. Optical density was test at 450nm. All experiments were performed in triplicate.

5-ethynyl-20-deoxyuridine (EdU) incorporation assays

MHCC-97H, SK-Hep1 and Huh7 cells seeded in 24-well plates were cultured in a humidified incubator for 12h. Cells were transiently transfected with vectors or siRNAs 12h later, according to the protocol. EdU Cell Proliferation Assay Kit (RiboBio, GUANZHOU, China) was used to detect the cellular proliferation after 48h. Briefly, fixed MHCC-97H, SK-Hep1 and Huh7 cells were stained with EdU following the recommendations after incubation with 50μM EdU. The ratio of EdU positive cells to total Hoechst positive cells was regarded as the EdU incorporation rate.

RNA interference

Small interfering RNAs were used in the current study to silence human CBX8 or YBX1 gene expression. All target sequences for CBX8 and YBX1 were synthesized by RiboBio Company (Ribobio, GUANZHOU, China). Randomly generated sequence, without sense, was used as a negative control (Scramble). Lipofectamine RNAiMAX Transfection Reagent (Invitrogen) was used to perform transfection assays.

Western blot

Total protein was extracted using radioimmuno precipitation (RIPA) assay buffer (Cell signaling Technology, Boston, MA, USA). The protein lysates were separated on a 12% SDS–polyacrylamide gel, and transferred onto polyvinylidene fluoride (Millipore, Bedford, MA, USA) membranes. After blocking with 5% BSA for 1 h at room temperature, the membranes were incubated with primary antibodies overnight at 4 °C. After washing, the membranes were incubated with the corresponding secondary antibodies conjugated to horseradish peroxidase. The membrane signals were detected using a commercial Excellent Chemi-luminescent Substrate (ECL) kit (Pierce, Rockford, IL, USA). ImageJ software was used to analyze the band intensity of western blotting and the normalization. The primary antibodies are shown in Supplementary Table 1.

Co-immunoprecipitation (Co-IP)

Proteins were extracted using immunoprecipitation assay buffer supplemented with a proteinase inhibitor cocktail. Primary antibody (anti-Flag: 5 μg/ml) was added to the lysate for 2.5 h. Protein A/G beads were added and the lysates incubated for an additional 2 h. Precipitated proteins were dissolved in sodium dodecyl sulfate (SDS) loading buffer and fractionated by SDS polyacrylamide gel electrophoresis.

Gene set enrichment analysis (GSEA)

GSEA was performed to confirm which gene sets or signatures were correlated with CBX8 or YBX1 expression in the TCGA dataset. The genome-wide expression profiles, including 372 samples, were downloaded from the TCGA dataset. Among these samples, a total of 351 samples with CBX8 or YBX1-expression data were entered for GSEA after being split into two groups (CBX8 low- or high-expression groups; YBX1 low- or high-expression groups). The GSEA software.

(GSEA v. 2.0, http://www.broadinstitute.org/gsea) was utilized to test whether members of the gene sets or signatures were randomly distributed at the top or bottom of the ranking (genes from patients were ranked based on the correlation between CBX8 or YBX1 expression). Once most members of a gene set were positively correlated with low expression of CBX8 or YBX1, the gene set was considered as having been correlated.

Real time quantitative polymerase chain reaction (qRT-PCR)

TRIzol® reagent (Invitrogen) was used for the extraction of total RNAs. First-strand cDNA was synthesized by the PrimeScript™ 1st Strand cDNA Synthesis Kit (TaKaRa, Tokyo, Japan). Real-time PCR was carried out using SYBR® Green PCR kit (TaKaRa). Primers are shown in Supplementary Table 2.

Lentiviral construction and cell transfection

The sh-CBX8 was obtained from Genechem Company Ltd. (Shanghai, China) and was used to knockdown CBX8 expression. A negative control lentiviral vector containing non-silencing short hairpin RNA was used. Huh7, MHCC-97H, and SK-Hep-1 cells were infected with either the lentiviral vectors encoding specific short hairpin RNA sequences or the negative control vector. The efficiencies of RNA interference were determined by western blot.
