# Supplementary material for: Chromobox homolog 8 (CBX8) Interacts with Y-Box binding protein 1 (YBX1) to promote cellular proliferation in hepatocellular carcinoma cells
Source: Aging (Albany NY). 2019 Sep 8;11(17):7123–49. doi: 10.18632/aging.102241 (PMC6756871; doi:10.18632/aging.102241)
Supplement: Supplementary Figures [file aging-11-102241-s003.pdf]

SUPPLEMENTARY FIGURES

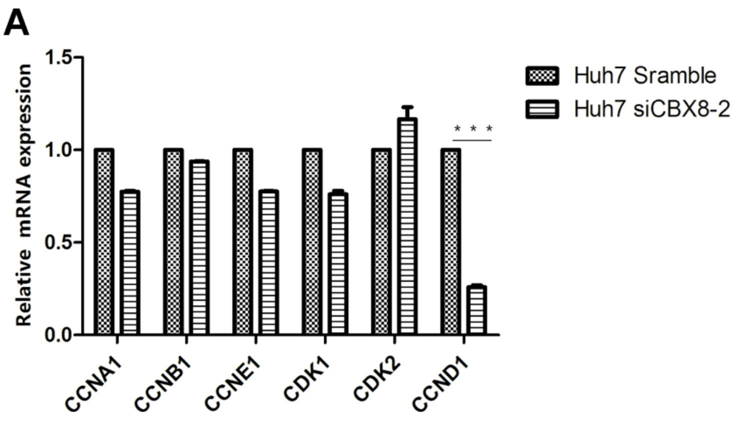

Supplementary Figure 1. CBX8 positively correlates with the cell cycle signaling pathway. (**A**) Cell cycle-related gene mRNA levels in CBX8-knockdown Huh7 cells, as detected by q-RT-PCR.

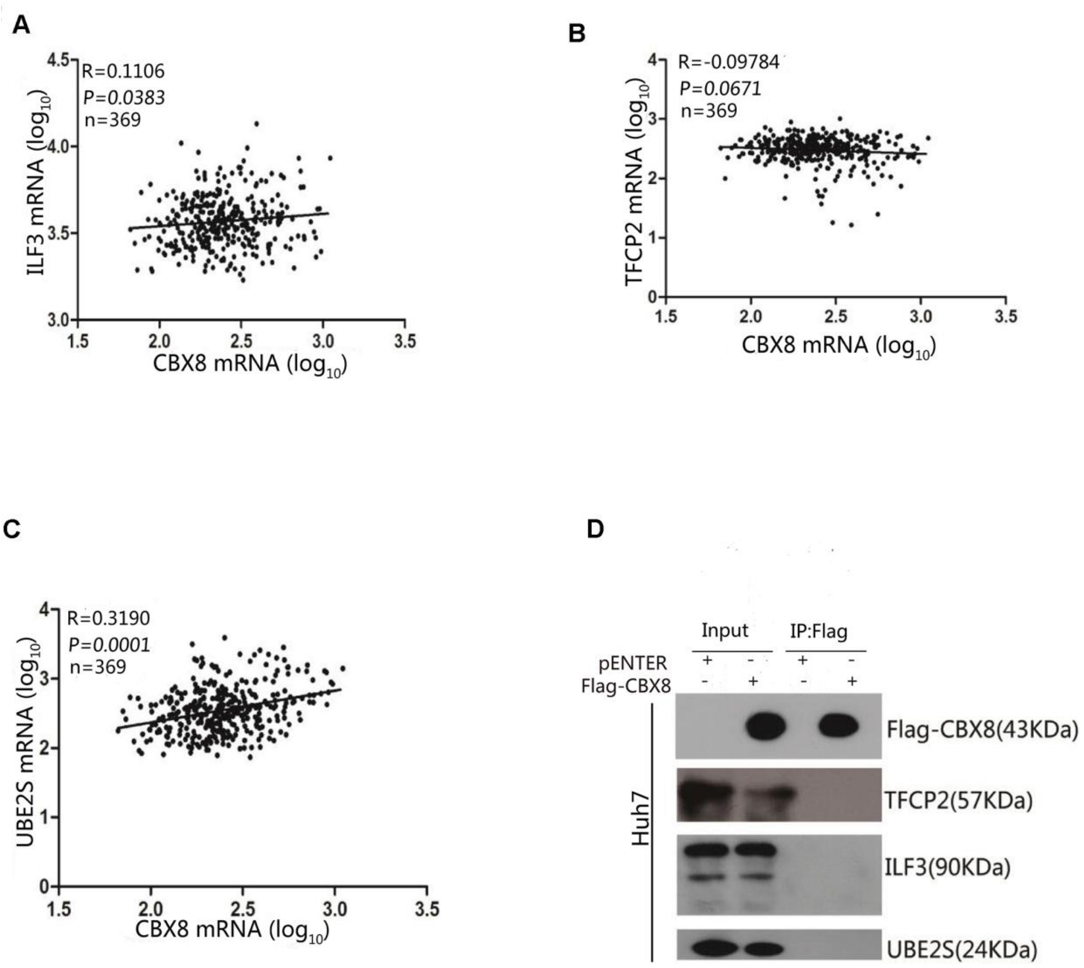

Supplementary Figure 2. CBX8 interact with YBX1. (**A**–**C**) Correlation between CBX8 mRNA and ILF3, TFCP2 and UBE2S mRNA in TCGA cohort. (**D**) Huh7 Cells were transfected with Flag-CBX8 overexpression vector for 48 h. An IP assay, using anti-Flag antibody, was used to detect the binding of CBX8 and ILF3, TFCP2, UBE2S.

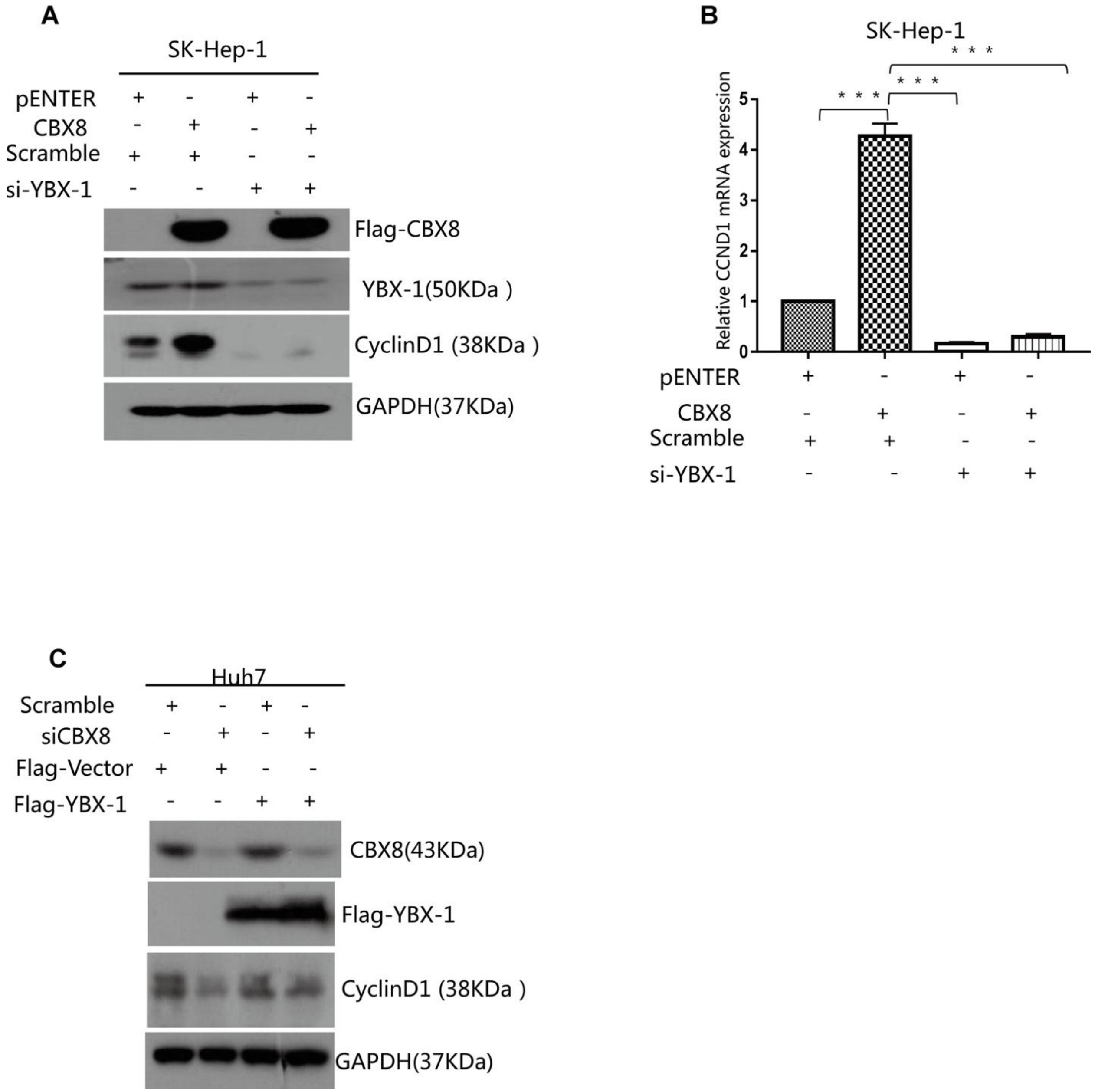

Supplementary Figure 3. CBX8 increase levels of CyclinD1 through YBX1. (**A**–**B**) Both protein and mRNA levels of CyclinD1 in CBX8-overexpressing or YBX1-knock-down SK-Hep1 cells, as detected by western blotting and q-RT-PCR. (**C**) Protein levels of CyclinD1 in CBX8-knock-down or YBX1-overexpressing Huh7 cells as detected by western blotting.

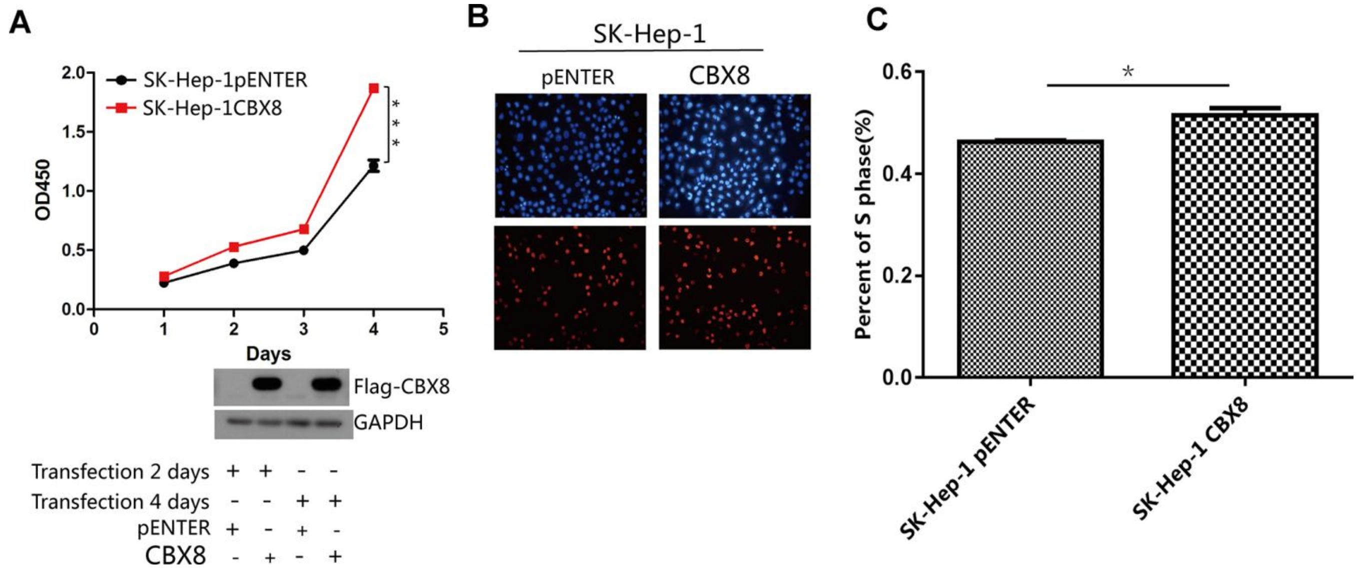

Supplementary Figure 4. CBX8 overexpression promotes cell proliferation. (**A**) Cell proliferation was evaluated by the CCK-8 assay. (**B**–**C**) Effect of CBX8 on cell proliferation, as measured by EDU assays **P* < 0.05; ***P* < 0.01; ****P* < 0.001.

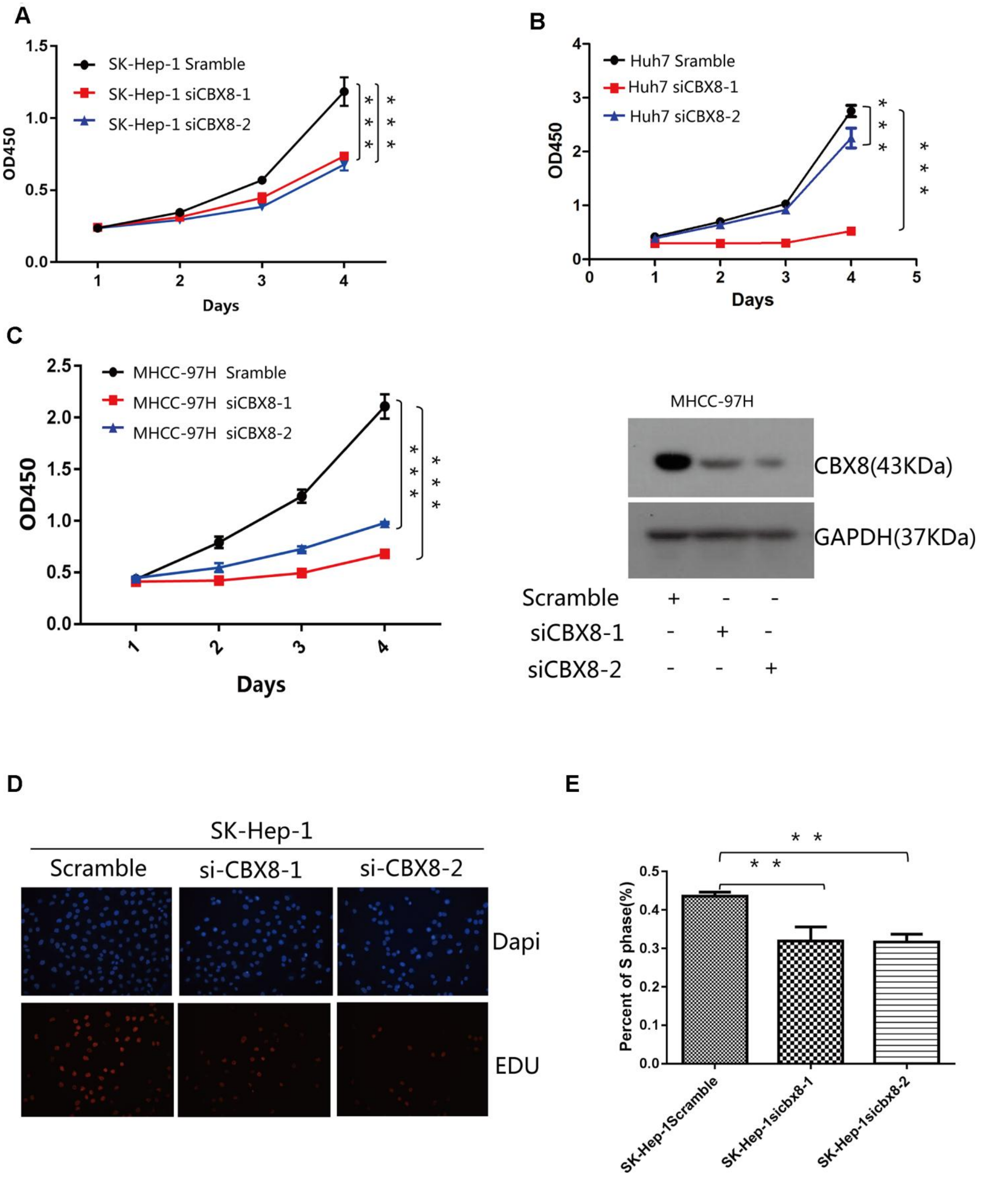

Supplementary Figure 5. Knockdown of CBX8 dramatically decreases cell proliferation in vitro. (**A**–**C**) Cell proliferation was evaluated by the CCK-8 assay. (**D**–**E**) Effect of CBX8 on cell proliferation, as measured by EdU assays *$P < 0.05$; **$P < 0.01$; ***$P < 0.001$.

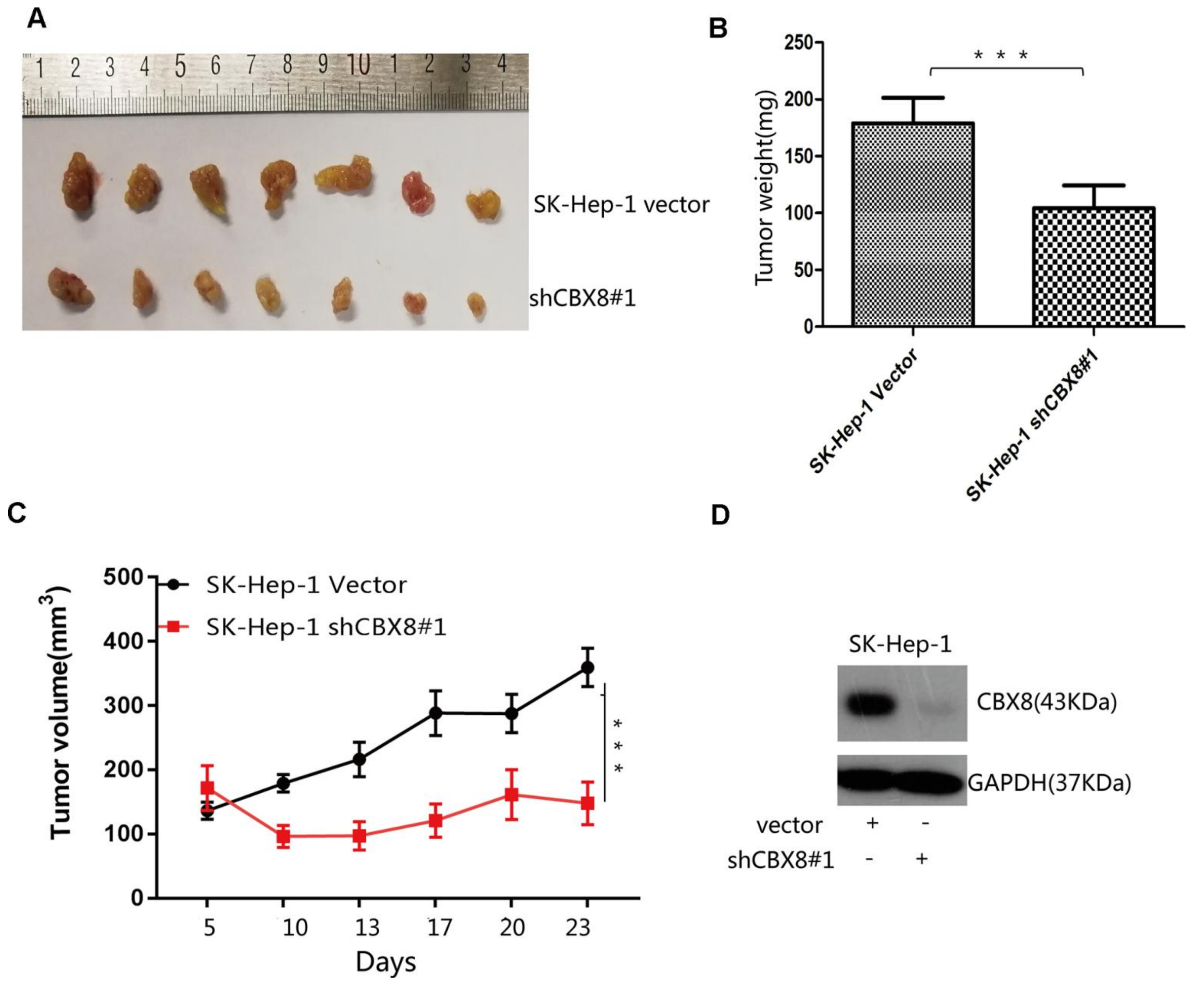

Supplementary Figure 6. Knockdown of CBX8 dramatically decreases cell proliferation in vivo (**A**) Representative images of tumors formed in nude mice injected subcutaneously with CBX8–silenced SK-Hep-1 cells. (**B**) Tumor weights (***P <0.001). (**C**) Tumors induced by CBX8 silencing in SK-Hep-1 cells (***P <0.001) showed markedly lower growth rates than control cells.

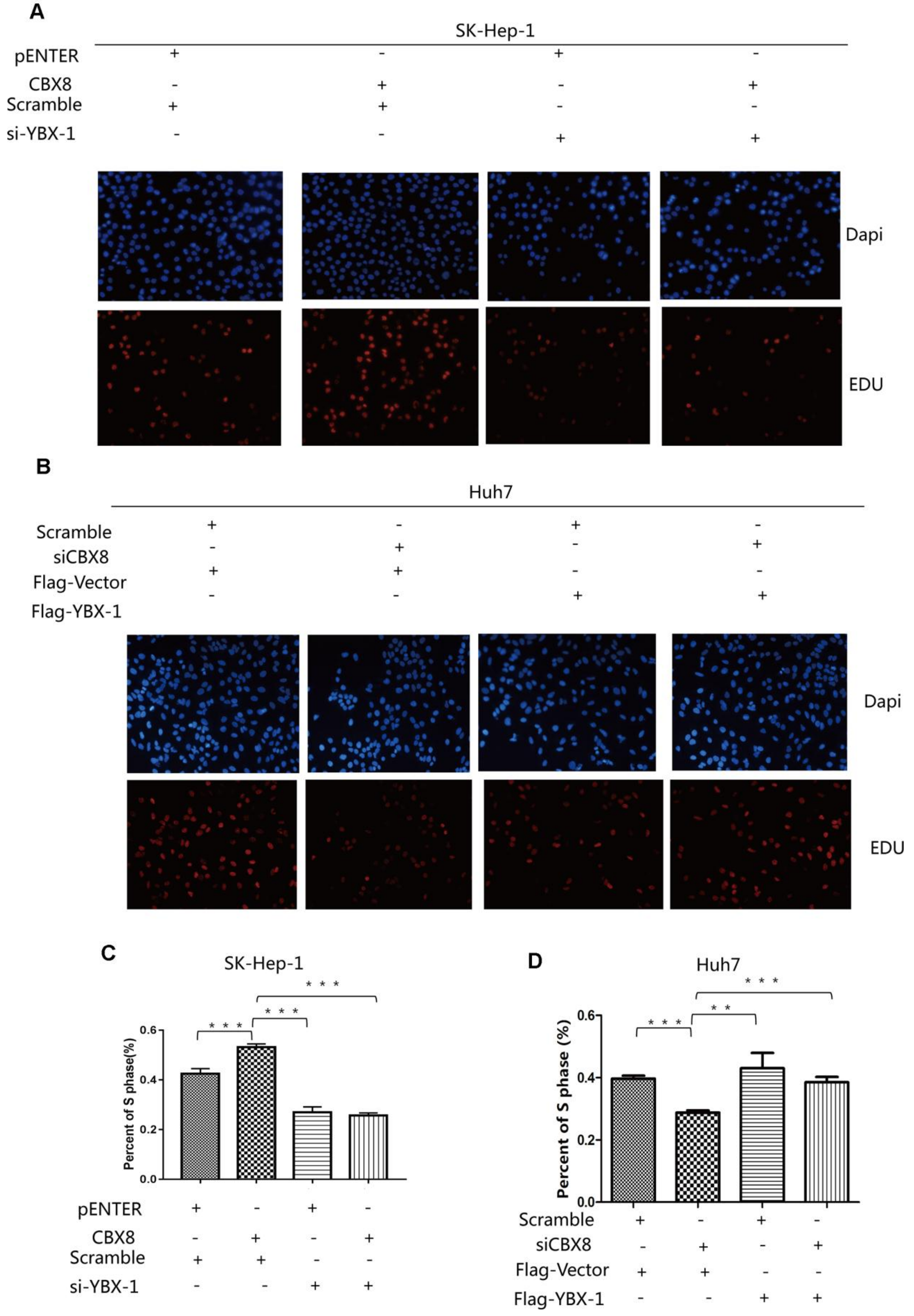

Supplementary Figure 7. CBX8 promotes HCC cell proliferation through YBX1. (**A**–**D**) Cell proliferation, as detected by EdU assays ***P*< 0.01; ****P* < 0.001.

A

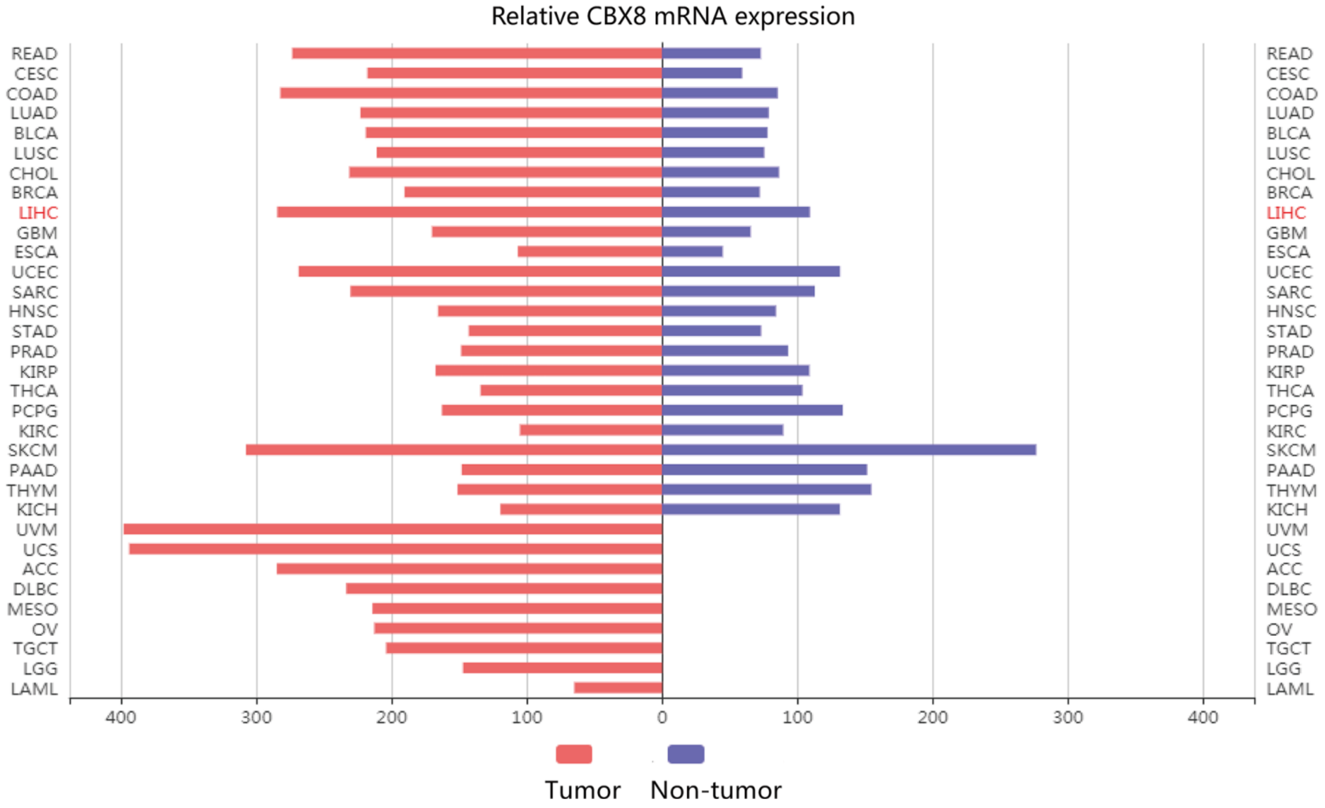

Supplementary Figure 8. CBX8 expression in human cancers (**A**) Relative CBX8 mRNA expression determined by RNA sequencing in human cancers from TCGA data (http://cancergenome.nih.gov/) using the GCBI website (https://www.gcbi.com.cn).

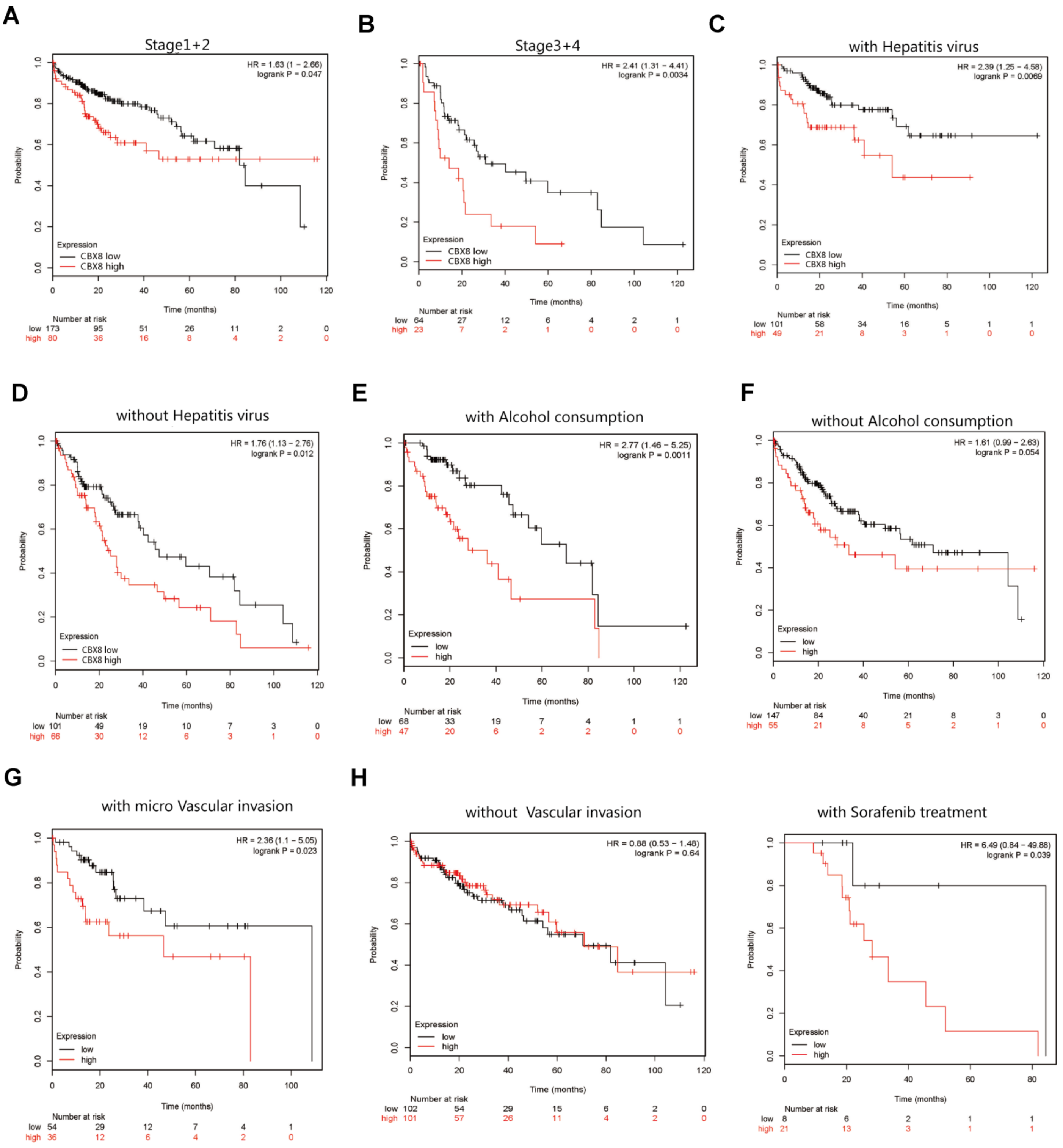

Supplementary Figure 9. The prognostic value of CBX8 in HCC. (**A**–**I**) Stratified analysis showed the correlation of CBX8 expression and overall survival in indicated groups in HCC from TCGA data using the Kaplan Meier-plotter website (http://kmplot.com/analysis/).

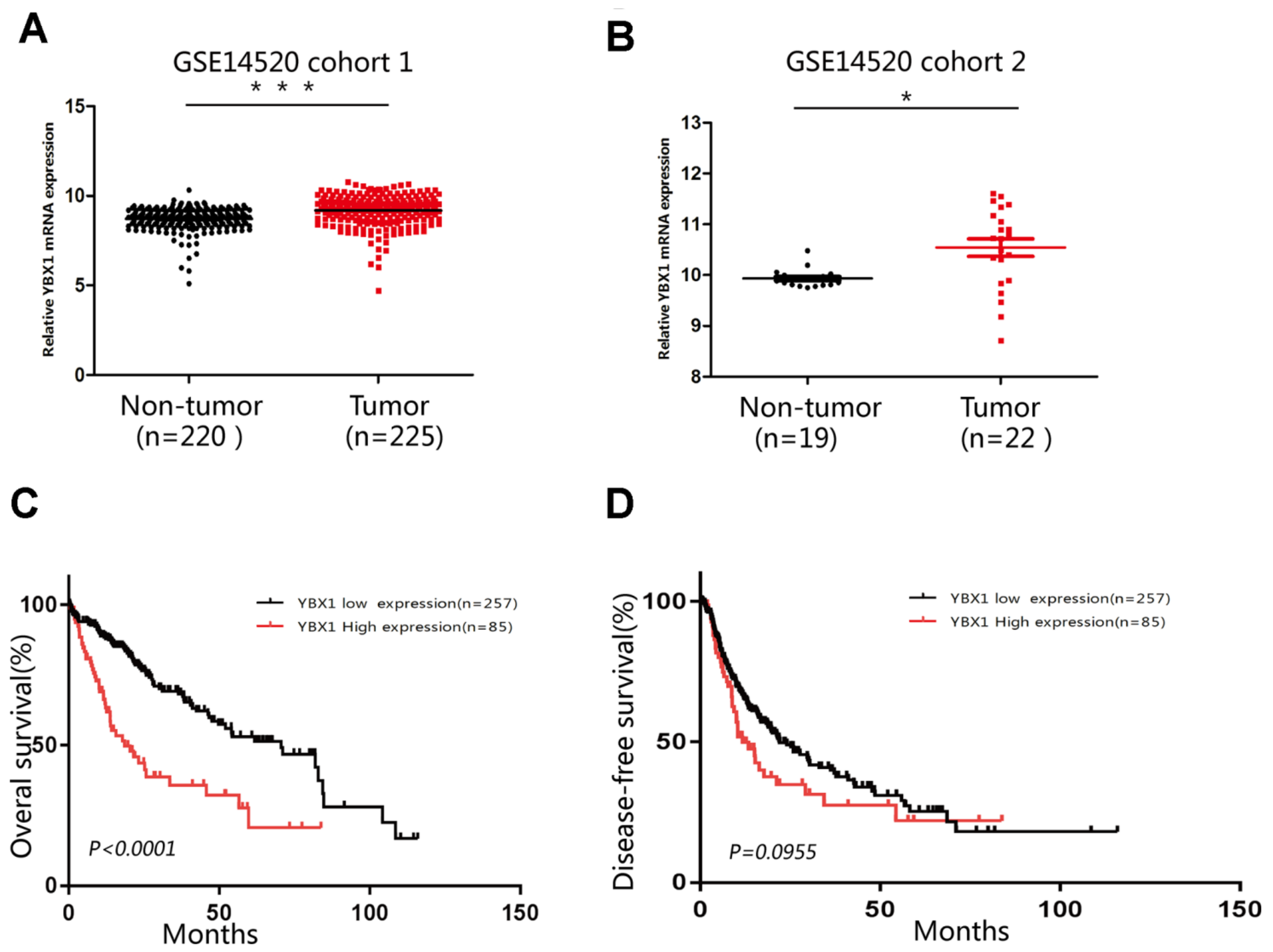

Supplementary Figure 10. YBX1 expression is up-regulated in HCC and is correlated with prognosis. (**A**–**B**) Expression of YBX1 in the GSE14520 cohort1 (P < 0.001) and GSE14520 cohort2 (P < 0.05). (**C**, **D**) Kaplan-Meier analysis of the overall and disease-free survival in the TCGA cohort based on YBX1 expression.

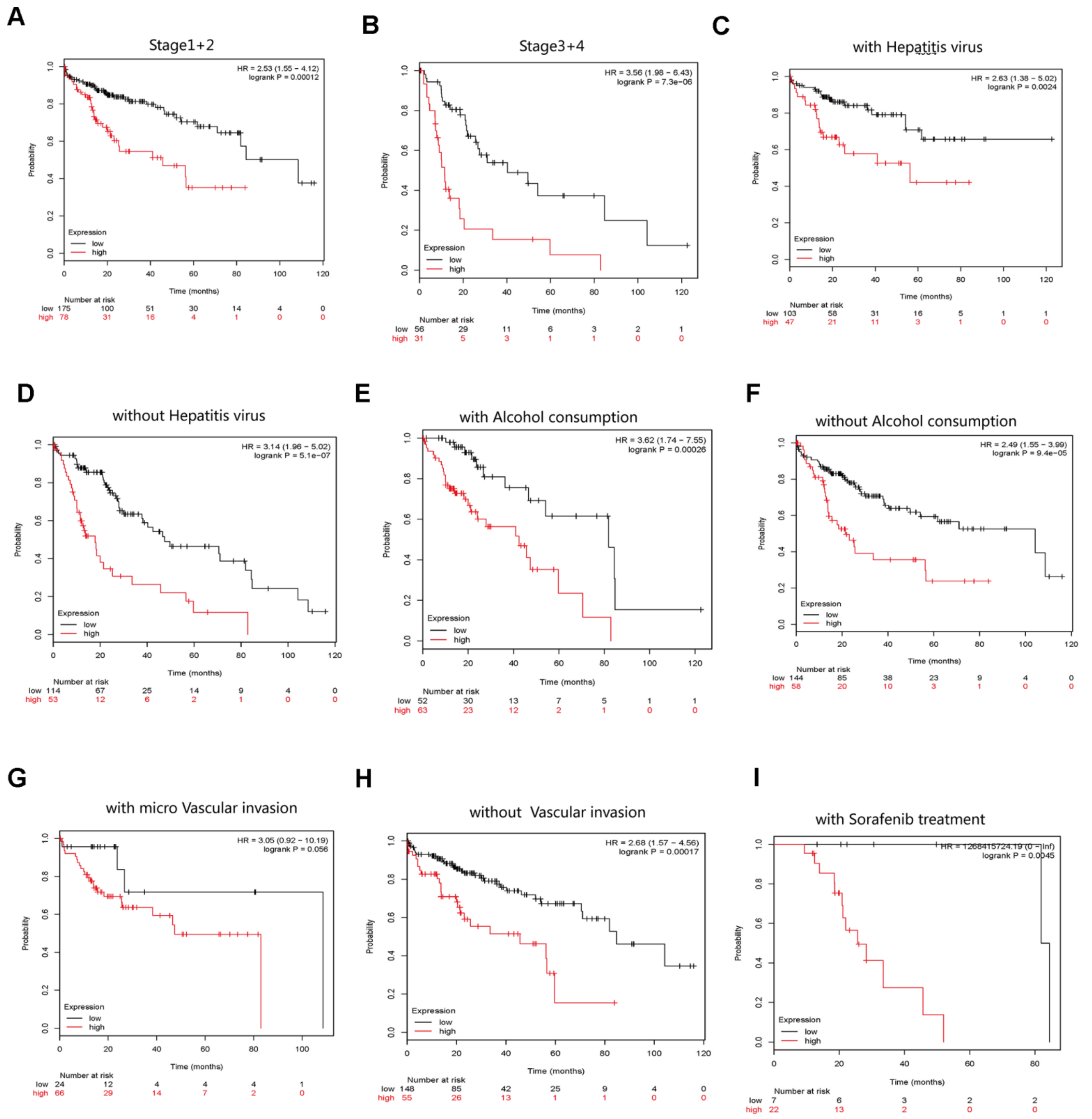

Supplementary Figure 11. The prognostic value of YBX1 in HCC. (**A**–**I**) Stratified analysis showed the correlation of YBX1 expression and overall survival in indicated groups in HCC from TCGA data using the Kaplan Meier-plotter website (http://kmplot.com/analysis/).
