# Supplementary material for: Chromobox homolog 8 (CBX8) Interacts with Y-Box binding protein 1 (YBX1) to promote cellular proliferation in hepatocellular carcinoma cells
Source: Aging (Albany NY). 2019 Sep 8;11(17):7123–49. doi: 10.18632/aging.102241 (PMC6756871; doi:10.18632/aging.102241)
Supplement: Supplementary Tables [file aging-11-102241-s002.pdf]

SUPPLEMENTARY TABLES

Supplementary Table 1. Information on antibodies used in this study.

Antibody	WB	specificity	Company
CBX8 (ab70796)	1:1000	Rabbit Polyclonal	Abcam
YBX1 (20339-1-AP)	1:500	Rabbit Polyclonal	Proteintech
Flag (F1804)	1:200	Mouse monoclonal	Sigma
CyclinD1 (SC8396)	1:200	Mouse monoclonal	Santa Cruz Biotechnology
UBE2S (14115-1-AP)	1:500	Rabbit Polyclonal	Proteintech
ILF3 (sc-136197)	1:200	Mouse monoclonal	Santa Cruz Biotechnology
TFCP2 (A5555)	1:500	Rabbit Polyclonal	ABclonal
β-actin(AC026)	1:7500	Mouse monoclonal	ABclonal
GAPDH (RM2002)	1:7500	Mouse monoclonal	Beijing Ray Antiboby Biotech

Supplementary Table 2. Primer sequence used in this study.

Gene	sequence
CBX8-F	5′ACGGAAAGGACGCATGGAAT 3′
CBX8-R	5′ CTTGGGTCCACGCTTTTTGG 3′
CCND1-F	5′ATGCCAACCTCCTCAACGAC 3′
CCND1-R	5′TCTGTTCCTCGCAGACCTCC3′
β-actin-F	5′ACAGAGCCTCGCCTTTGCC 3′
β-actin-R	5′ GATATCATCATCCATGGTGAGCTGG3′
CCNA1-F	5′ GAGAACGGGTCACGGAAACA3′
CCNA1-R	5′ACTGTAGCCAGCACAACTCC3′
CCNB1-F	5′GAAACGCATTCTCTGCGACC3′
CCNB1-R	5′ ACACCCAGCAGAAACCAACA3′
CCNE1-F	5′GCAGGATCCAGATGAAGAAATG3′
CCNE1-R	5′TAATCCGAGGCTTGCACGTT3′
CDK2-F	5′CCGAGCTCCTGAAATCCTCC3′
CDK2-R	5′CCCAGAGTCCGAAAGATCCG3′
CDK1-F	5′TTTCTTTCGCGCTCTAGCCA3′
CDK1-R	5′CAATCGGGTAGCCCGTAGAC3’
